# Automatic dental age calculation from panoramic radiographs using deep learning: a two-stage approach with object detection and image classification

**DOI:** 10.1186/s12903-024-03928-0

**Published:** 2024-01-31

**Authors:** Kazuma Kokomoto, Rina Kariya, Aya Muranaka, Rena Okawa, Kazuhiko Nakano, Kazunori Nozaki

**Affiliations:** 1 Division for Medical Informatics, Osaka University Dental Hospital, 1-8 Yamada-oka, Suita, Osaka, 565-0871 Japan; 2 Department of Pediatric Dentistry, Osaka University Graduate School of Dentistry, 1-8 Yamada-oka, Suita, Osaka, 565-0871 Japan

**Keywords:** Artificial intelligence, Machine learning, Medical informatics applications, Pediatric dentistry, Orthodontics, Dental informatics

## Abstract

**Background:**

Dental age is crucial for treatment planning in pediatric and orthodontic dentistry. Dental age calculation methods can be categorized into morphological, biochemical, and radiological methods. Radiological methods are commonly used because they are non-invasive and reproducible. When radiographs are available, dental age can be calculated by evaluating the developmental stage of permanent teeth and converting it into an estimated age using a table, or by measuring the length between some landmarks such as the tooth, root, or pulp, and substituting them into regression formulas. However, these methods heavily depend on manual time-consuming processes. In this study, we proposed a novel and completely automatic dental age calculation method using panoramic radiographs and deep learning techniques.

**Methods:**

Overall, 8,023 panoramic radiographs were used as training data for Scaled-YOLOv4 to detect dental germs and mean average precision were evaluated. In total, 18,485 single-root and 16,313 multi-root dental germ images were used as training data for EfficientNetV2 M to classify the developmental stages of detected dental germs and Top-3 accuracy was evaluated since the adjacent stages of the dental germ looks similar and the many variations of the morphological structure can be observed between developmental stages. Scaled-YOLOv4 and EfficientNetV2 M were trained using cross-validation. We evaluated a single selection, a weighted average, and an expected value to convert the probability of developmental stage classification to dental age. One hundred and fifty-seven panoramic radiographs were used to compare automatic and manual human experts’ dental age calculations.

**Results:**

Dental germ detection was achieved with a mean average precision of 98.26% and dental germ classifiers for single and multi-root were achieved with a Top-3 accuracy of 98.46% and 98.36%, respectively. The mean absolute errors between the automatic and manual dental age calculations using single selection, weighted average, and expected value were 0.274, 0.261, and 0.396, respectively. The weighted average was better than the other methods and was accurate by less than one developmental stage error.

**Conclusion:**

Our study demonstrates the feasibility of automatic dental age calculation using panoramic radiographs and a two-stage deep learning approach with a clinically acceptable level of accuracy.

## Introduction

Growth and development assessment in children is essential for making appropriate diagnosis and treatment decisions in orthodontic and pediatric dentistry [1]. Dental age and chronological age are different ways of measuring a person’s developmental stage. Chronological age refers to a person’s actual age based on their date of birth. This is the most commonly used way of measuring age and is used to determine when a person reaches certain developmental stages. Dental age, on the other hand, is an estimate of a person’s age based on the development of their teeth. While chronological age is just a fixed number that does not change, dental age can vary depending on a person’s growth and thus provide more important and individual information. In addition, dental age is useful in deciding when to initiate orthodontic treatment or whether a child’s dental development is delayed or advanced since chronological age is not always equal to dental age [2, 3].

The various dental age calculation methods can be categorized as morphological methods, biochemical methods, and radiological methods [4, 5]. Morphological methods are based on measurement of actual teeth and regression formulas are used for calculation. Biochemical methods are based on the racemization of amino acids [6]. Radiological methods are commonly used since they are non-invasive and reproducible compared to other methods [7–11]. Estimating dental age is feasible when radiographs are available, by assessing the growth stage of permanent teeth and converting this stage into an estimated age using a lookup table, or by measuring the distance from landmarks such as the tooth, root, or pulp, and inputting these values into regression equations [4, 7].

However, these processes are predominantly manual and require considerable time. The average time for manually calculating dental age is 10 min [12], making it inconvenient to be performed for every patient in daily clinical practice. Therefore, automatic dental age calculation is expected to save time in treatment planning by eliminating time-consuming but crucial routine tasks and increasing the interaction time between dentists and patients [13].

Recently, deep learning with Convolutional Neural Networks (CNN) and Artificial Intelligence (AI) in computer vision has been developed, which can automatically extract imaging features with original pixel information as input data. In previous studies, deep learning methods for object detection, image classification, and image segmentation were widely used in dentistry [14–19]. Furthermore, the field of chronological age calculation has seen a growing interest in applying these techniques [20–23]. Studies have demonstrated that CNN models can surpass the accuracy of manual methods in classifying chronological age based on dental images. However, few studies included multiple deep learning techniques and addressed germ detection or developmental stage classification. In addition, those methods have been developed for chronological age calculation, not for dental age calculation.

This study aimed to fill the gaps between classical manual calculations and modern AI technologies in the field of dental age calculation. We proposed a novel and completely automatic dental age calculation method using panoramic radiographs and two-stage deep learning combined with object detection and image classification, trained with voluminous images. Additionally, we evaluated its accuracy by comparing automatic and expert manual calculations and whether our proposed method could be clinically acceptable.

## Materials and methods

### Dataset

**Fig. 1:**
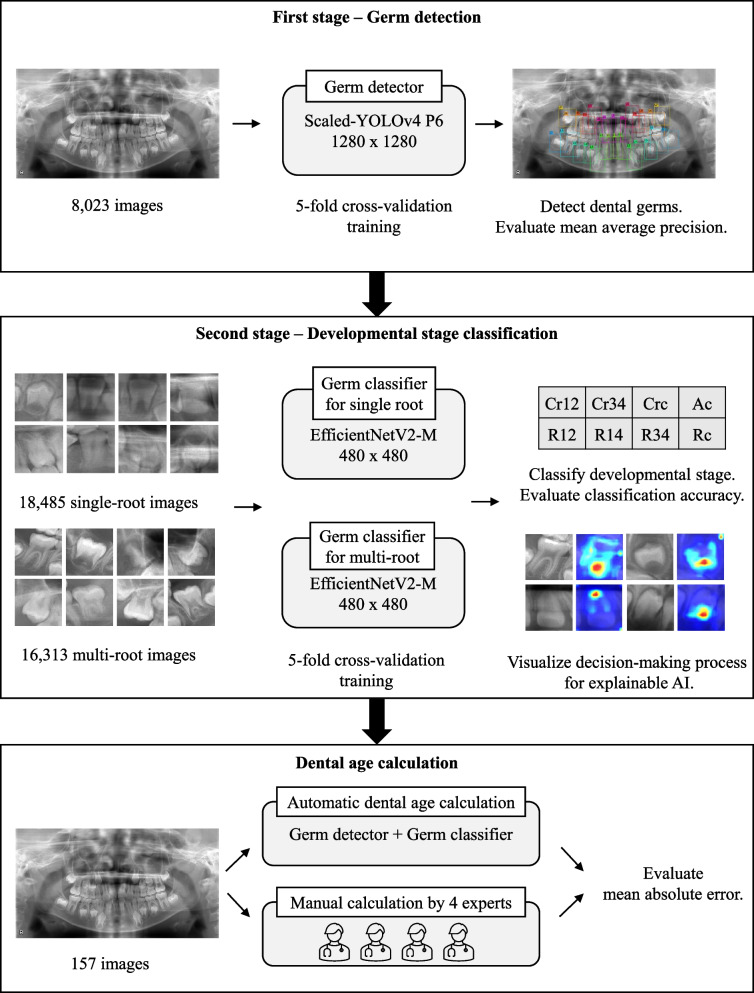
Our pipeline processes for automatic dental age calculation and evaluation methods

This study was retrospective and observational in nature. All images used in this study were obtained from patients who received dental treatment between January 2000 and December 2018 at Osaka University Dental Hospital, Department of Pediatric Dentistry, Osaka, Japan. All images were anonymized and had no metadata such as patient name, chronological age, sex, dentition, or disease due to ethics. Roughly speaking, the datasets contained a relatively high number of images that showed healthy dentition. Our proposed process is illustrated in Fig. 1.

### Germ detection

We utilized Scaled-YOLOv4 [24] as our germ detector, which is an improved version of YOLOv4 [25] and has achieved state-of-the-art object detection. Scaled-YOLOv4 has performed well with larger models and input image sizes [24]; therefore, we used the second largest model, Scaled-YOLO v4 P6, for germ detection with an input size of 1280 × 1280 pixels, as much as our computational resource allowed.

To train Scaled-YOLOv4, 8023 panoramic radiographs were used as training data. These images included primary, mixed, and permanent dentitions. Four pediatric dentists were presented with panoramic radiographs and instructed to draw box boundaries around all dental germs and class-label those boxes. The class labels were based on a palmer system [26]. The correspondence between number and tooth; 1 was the central incisor, 2 was the lateral incisor, 3 was the canine, 4 was the first premolar, 5 was the second premolar, 6 was the first molar, 7 was the second molar, and 8 was the third molar. We added upper case prefixes “U” and “L” to identify the upper and lower tooth, respectively. For instance, U1 denotes the upper central incisor, and L6 denotes the lower first molar.

Our model’s performance was evaluated using average precisions with a 0.5 Intersection over Unit threshold, which is a common metric in object detection deep learning models, including the YOLO families [24, 25, 27–31], known as AP_50_. We performed the 5-fold cross-validation to assess the generalization of our model and prevent overfitting [32]. The training datasets were split into five, and Scaled-YOLOv4 was trained using four of them. The remaining fold was used to calculate the AP_50_ of the trained model. We replicated this five times and evaluated the average of the out-of-fold predictions.

### Developmental stage classification

In total, 18,485 single- and 16,313 multi-root dental germ images were prepared using our germ detector and used as the training dataset. Four pediatric dentists were presented with dental germ images and instructed to classify developmental stages in Japanese children as Cr1/2 (1/2 crown formation), Cr3/4 (3/4 crown formation), Crc (crown complete), R1/4 (1/4 root formation), R1/2 (1/2 root formation), R3/4 (3/4 root formation), Rc (complete root formation), and Ac (apex closed) [8]. Ci (initial calcification) and Cco (coalescence of cusps) were excluded because the images were too few to train the model. Each of the four pediatric dentists annotated the distinct images once and did not re-annotate any image annotated by the other dentists.

We utilized EfficientNetV2 to classify dental germ images [33]. EfficietNetV2 is an improved version of EfficientNet [34] and a CNN-based image classification model that achieves state-of-the-art performance on the ImageNet dataset with better accuracy and efficiency than previous famous models, such as ResNet [35], DenseNet [36], and Xception [37]. EfficientNetV2 scales up from EfficientNetV2-S to EfficientNetV2-M/L, and classification performance also improves as the model scales up. However, the computational complexity increases exponentially as EfficientNetV2 scales up; therefore, we chose the intermediate EfficientNetV2-M as our germ classification model. All germ images were resized to 480 × 480 pixels to train EfficientNetV2-M. We performed 5-fold stratified cross-validation so that each fold could have the same proportion of developmental stages. The classification accuracy of out-of-fold predictions was evaluated using the same procedure as germ detection. We evaluated the Top-1 accuracy and Top-3 accuracy. The former is a metric of model prediction performance that must match the single developmental stages. The latter is the classification correctness, in which the top three highest probabilities of model predictions matched the target developmental stages.

It is also crucial to know the decision-making process of AI for interpretability and explainability [38, 39]. We applied Gradient-weighted Class Activation Mapping (Grad-CAM) [40] to analyze how our model could classify dental germs and whether the procedure was similar to that used by dentists.

### Dental age calculation

In the germ classification stage, we obtained the probability of each dental germ’s developmental stage. We evaluated a single selection, a weighted average, and an expected value to convert the probability to dental age. The Ac stage was excluded from calculation because this stage refers to the end of dental germ development and does not have a dental age [8]. The single selection chooses one developmental stage with the highest probability and converts it to the respective dental age [8]. The weighted average considers the probability of each developmental stage. We used the three highest probabilities to calculate the weighted average, as follows:

$$weighted\ average=\frac{x_1{p}_1+{x}_2{p}_2+{x}_3{p}_3}{p_1+{p}_2+{p}_3}$$

where *p*_1_, *p*_2_, *and p*_3_ are the top three probabilities of the dental germ’s developmental stage, as obtained using the germ classifier, and *x*_1_, *x*_2_, *and x*_3_ are the dental ages of the corresponding developmental stages. The expected value considers all probabilities and was calculated as follows:

$$expected\ value=\frac{\sum_{i=1}^n{x}_i{p}_i}{\sum_{i=1}^n{p}_i}$$

where *n* is the number of developmental stages used for calculation, *p*_*i*_ are the probabilities of the dental germ’s developmental stage, and *x*_*i*_ are the dental ages of the corresponding developmental stages.

After converting the probability to dental age, the simple average of the dental age of each dental germ was regarded as the overall dental age of each panoramic radiograph [41]. To analyze the accuracy of the overall dental age, 157 panoramic radiographs that were not included in previous training datasets were used, and automatic dental age calculation was performed using one of the 5-fold cross-validated germ detector and germ classifier models. Four pediatric dentists manually calculated the overall dental age using the same radiographs, and the mean absolute errors between the experts’ and the automatic calculation were evaluated. Since all the images used in this study were anonymized and had no metadata in terms of sex, we calculated dental age of males and females from the same panoramic radiograph and evaluated an average of both.

## Results

**Fig. 2:**
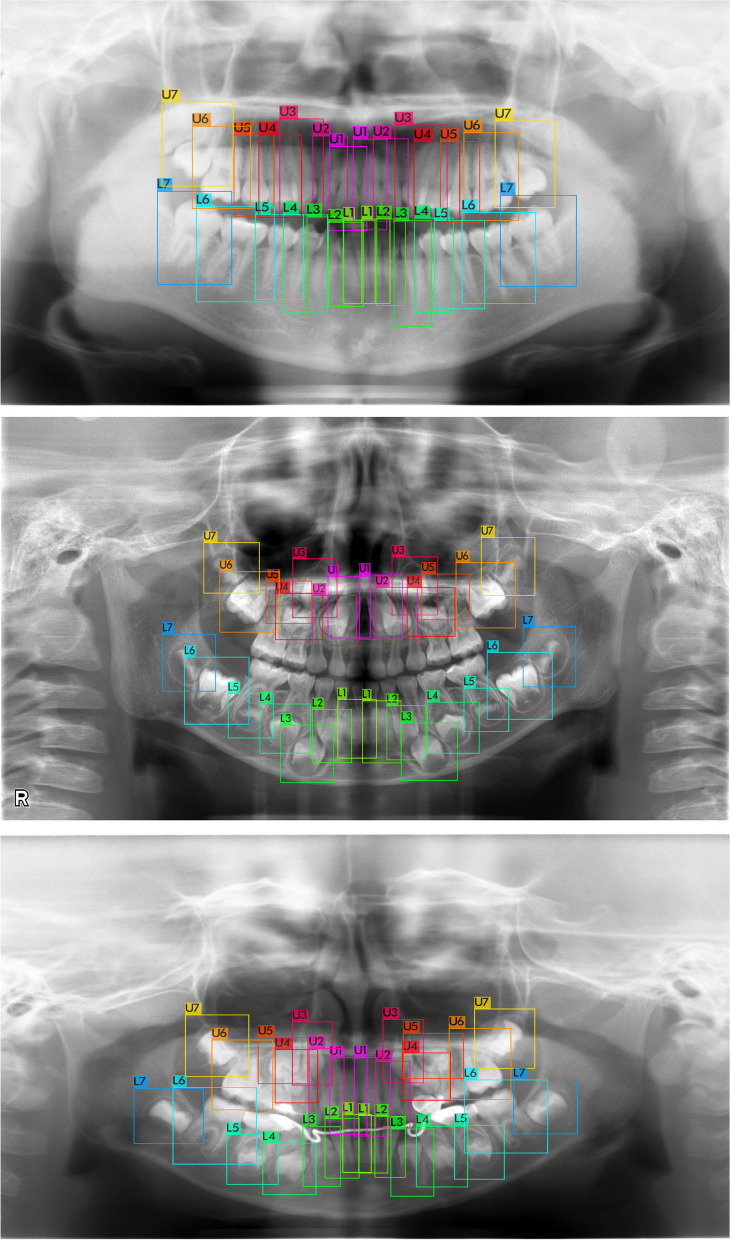
Examples of germ detection without congenitally missing teeth. All germs were detected correctly

**Fig. 3:**
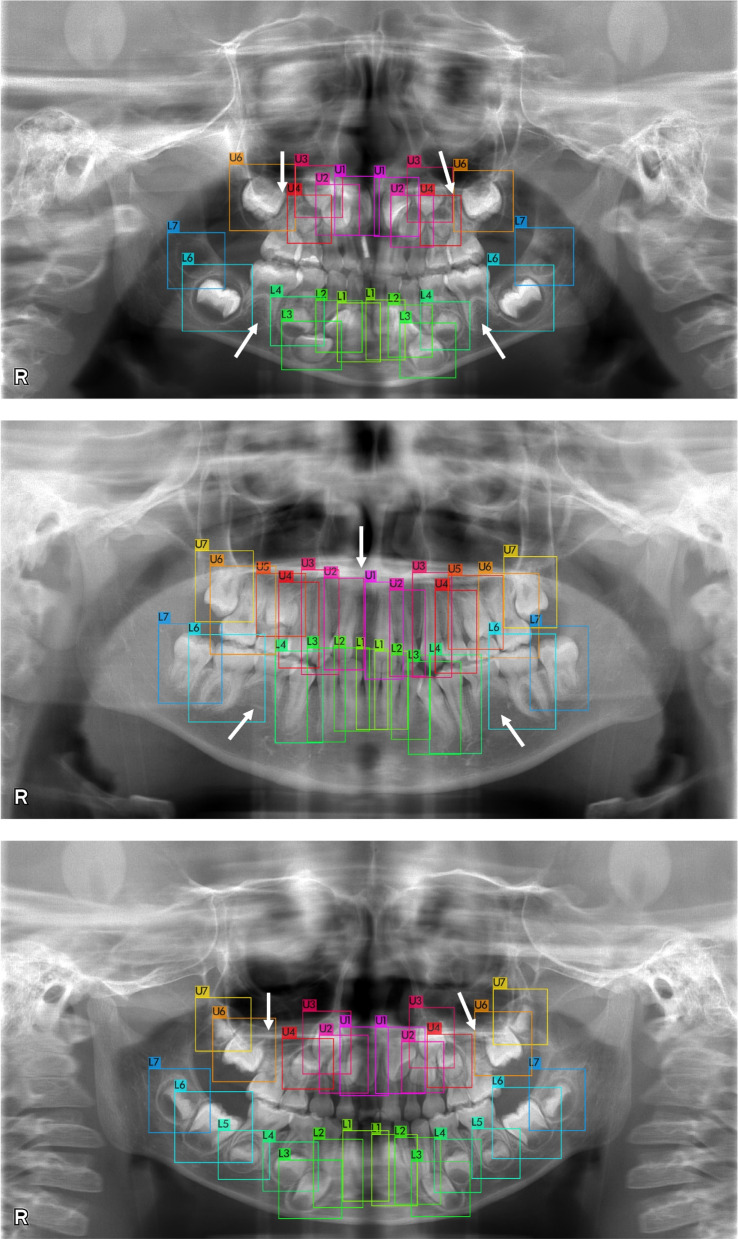
Examples of germ detection with congenitally missing teeth. Arrows indicate the points where dental germs are absent and not detected

**Fig. 4:**
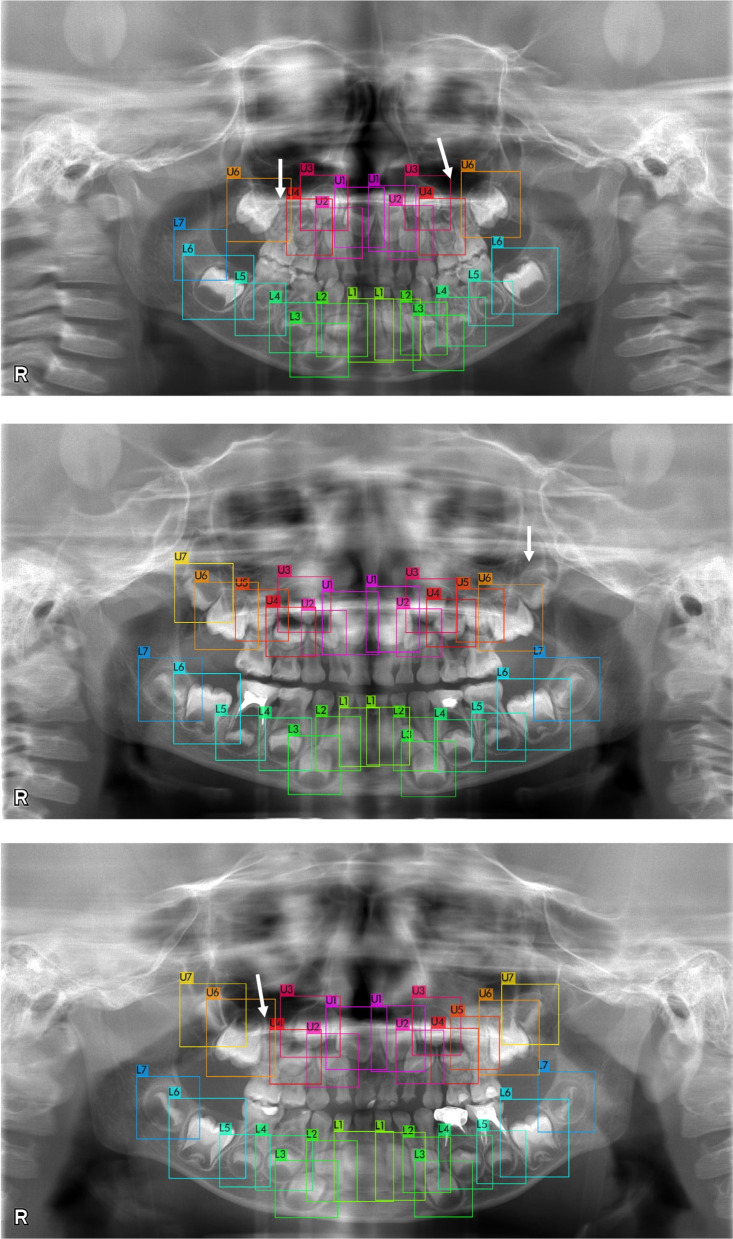
Examples of germ detection with some failure. Some germs were not detected. Arrows indicate points where dental germs are present but not detected

The germ detector’s performance is presented in Table 1. Scaled-YOLOv4 P6 with an input image size of 1280 × 1280 achieved detection accuracy with an AP_50_ of 98.26%. Examples of germ cell detection with and without congenitally missing teeth are presented in Figs. 2, 3, and 4. Notably, the dental germs were correctly detected. Primary tooth conditions, such as healthy, caries, composite filling, metal crown, root canal filling, and orthodontic materials, did not affect detection performance. Examples of germ detection failures are illustrated in Fig. 4. Most dental germs were accurately detected; however, some dental germs were not.

**Table 1:** Average precisions of our germ detector and accuracy of the developmental stage classifier

Fold	Germ detection	Developmental stage classification			
		Single-root		Multi-root	
	AP_50_	Top-1	Top-3	Top-1	Top-3
1	98.32	66.68	98.32	72.35	98.16
2	98.38	68.43	98.54	71.61	98.62
3	98.14	68.23	98.57	71.74	98.41
4	98.23	68.16	98.13	71.01	98.16
5	98.23	70.03	98.76	71.01	98.44
Average	98.26	68.30	98.46	71.54	98.36

**Fig. 5:**
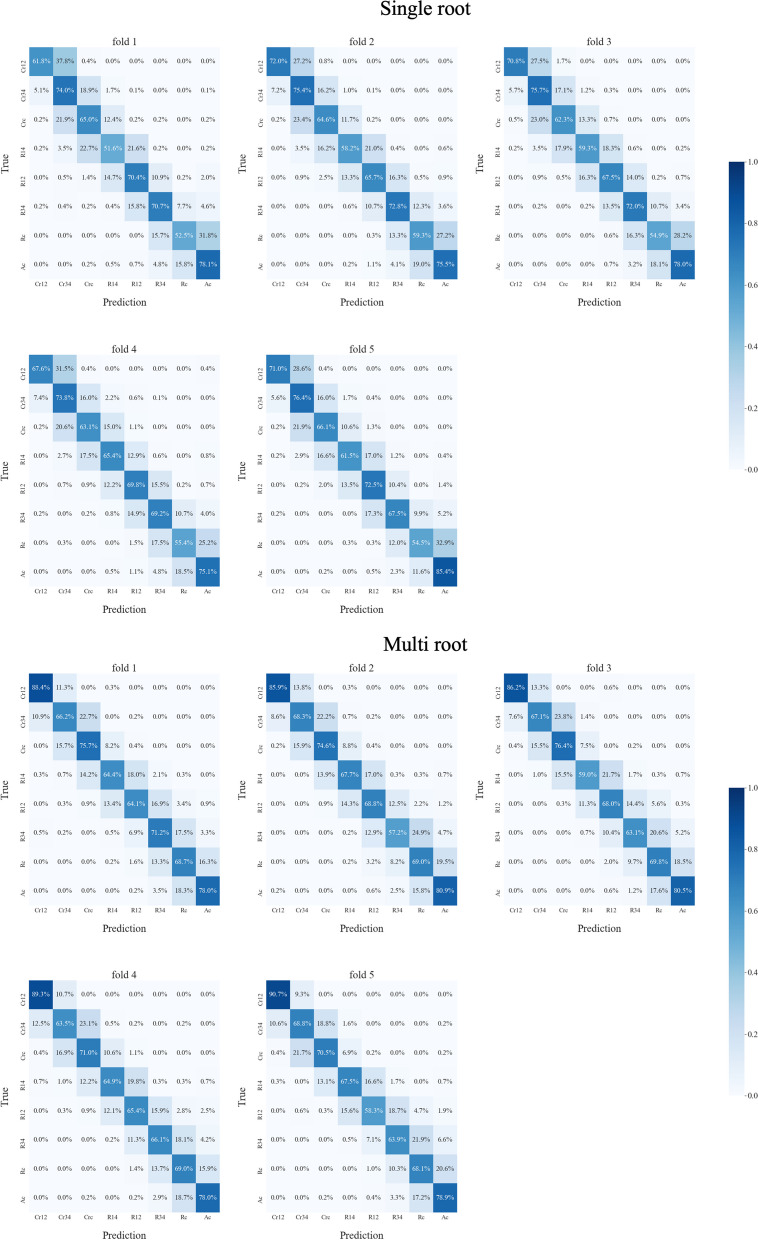
Results of germ classifier for single- or multi-root dental germ images obtained for each fold of cross-validation training. Those confusion matrices are normalized by the number of elements in each class to reveal each class’s accuracy

**Fig. 6:**
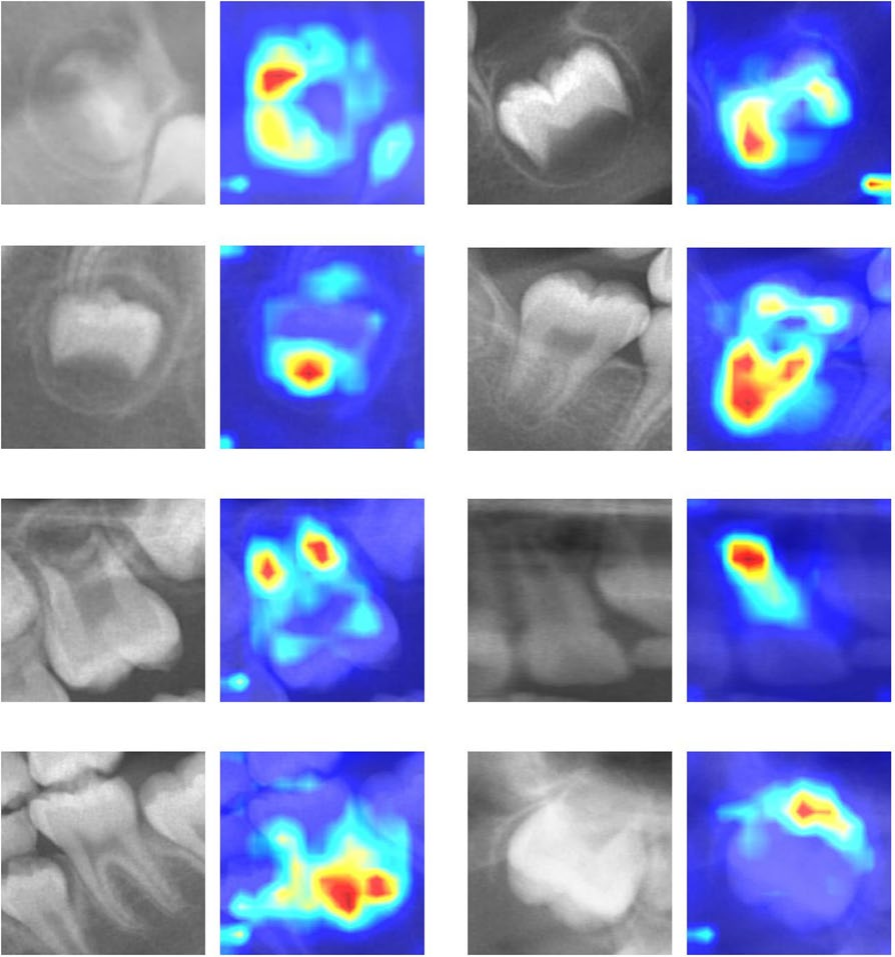
Examples of representative dental germ images and corresponding Grad-CAM of germ classifier for interpretability and explainability. Germ classifier can recognize the shape or form of the dental germ

The performance of the germ classifier for single- or multi-root dental germ images with 5-fold cross-validation is summarized in Table 1. The germ classifier for single- and multi-root dental germs achieved the highest Top-1 classification accuracies of 68.31 and 71.54% and the Top-3 accuracies of 98.46 and 98.36%, respectively. The confusion matrices for germ classification are presented in Fig. 5. The germ classifier tended to misclassify the actual stages as adjacent stages. The Grad-CAM images of the germ classifier are illustrated in Fig. 6. The germ classifier can recognize the shape or form of the dental germ like human experts.

The mean absolute errors between the automatic and manual overall dental age calculations by the four experts using single selection, weighted average, and expected value to convert the probability of each dental germ’s developmental stage to each dental age are described in Table 2. The weighted average was better than the other methods for the conversion to dental age.

**Table 2:** The mean absolute errors between the automatic and manual overall dental age calculations

Calculation method	Mean absolute error	Standard deviation
Single selection	0.274	0.342
Weighted average	0.261	0.274
Expected value	0.396	0.588

## Discussion

In this study, our dental germ detector using Scaled-YOLOv4 P6 with an input size of 1280 × 1280 achieved a very high AP_50_ of over 98% by cross-validation, as presented in Table 1. The training data, which was much larger than that of previous studies [14, 15], was sufficient for our model to learn the features of images. Generally, there were two choices for object identification from the image: semantic segmentation and object detection. Since pixel-level annotation for semantic segmentation was costly and erroneous and object detection was better at handling overlapping objects [19], we selected object detection for germ detection.

In addition, since the method of obtaining panoramic radiography with optimal quality has been established [42], our models could achieve high performance by learning dental germ features, including background images, overlapping with other objects, or relative position to other dental germs. Therefore, Scaled-YOLOv4 or older YOLO families [25, 27] may have sufficiently detected dental germs, and the newest, but computationally time-consuming models, such as YOLOv7 [43], were not necessary.

However, using state-of-the-art image models for dental germ-stage classification is important. Despite EfficientNet V2’s exceptional performance, the Top-1 accuracy of our germ classifier was approximately 70%, as presented in Table 1. This might be because overlapping with other objects or background images, thought to be good for germ detection models, negatively affects the germ classification model. Therefore, we utilized one of the state-of-the-art but computationally expensive models for germ classification. Our germ classification models are considered to be similar to human experts and are clinically applicable with reasonable accuracy. Our models focus on the crown shape or root formation of the dental germ to classify developmental stages, as illustrated in Fig. 6 like human experts. In addition, our model tends to misclassify adjacent stages using the confusion matrix in Fig. 5. This tendency was observed in previous research and also in real-world dentists because the adjacent stages of the dental germ looks similar and the many variations of the morphological structure can be observed between developmental stages [8, 44]. This is why we achieved an exceptional Top-3 accuracy of 98%. It is also the reason we adopted the Top-3 weighted averages to calculate dental age, which reduced the mean absolute error between the automatic and manual calculations by experts, as presented in Table 2. The single selection showed a similar mean absolute error, but the standard deviation was worse than the weighted average, indicating that the calculated value may spread out over a wide range and may be far from the actual dental age. The expected value showed the worst result, which suggested that using all data may be noisier than using Top-3 accurate values.

Our germ detection model achieved a high AP_50_ of 98%; nonetheless, a few dental germs were sometimes not detected, as illustrated in Fig. 4. However, this may not be critical as regards dental age calculation because we can still use over 20 dental germs and average them for calculation despite several germ detection failures. Thus, our automatic calculation method is robust against detection failures.

Our automatic dental age calculation achieved a mean absolute error of 0.261 years (about 3 months) compared with human experts, raising the concern of this difference being clinically acceptable. Most previous studies have focused on chronological age estimation [20–23, 45], whereas our research aimed to evaluate dental age calculation. Therefore, our results are not directly comparable to those of previous studies. One potential metric to evaluate our results can be the difference in years between the developmental stages of the teeth. For each teeth, the minimum difference between each developmental stage and its adjacent stage is 0.4 years [8]. Our model has achieved better result of 0.261 years, indicating that our automatic dental age calculation is accurate by less than one developmental stage error and thus is acceptable for supporting dentists. Moreover, the automatic calculation can be performed in a few minutes, which is significantly faster than manual calculation [12] and is useful not only for pediatric or orthodontic dentists but also for general dentists and even students. We believe that our results will serve as a new benchmark for further research in dental age calculation.

Our method can easily be applied to other dental age calculation methods based on developmental stage assessment [9–11]. For those methods, first, determine the dental germs should be determined. Then the developmental stages should be classified to obtain dental age, using the procedure described in our model. If another method is to be used, the model should be modified to change the calculation algorithm, including the volume assessment of teeth, pulp-to-tooth ratio method, coronal pulp cavity measurement, and open apices method [4, 5, 7].

Our proposed model can be useful not only for dental age calculation using various methods but also other clinically supportive applications. When there were congenitally missing teeth on the panoramic radiograph, the germ detector did not respond to the missing teeth’s location, as shown in Fig. 3. This behavior can inform dentists about missing teeth, which is a crucial factor in treatment planning. Moreover, our germ classifier can help human experts improve their diagnostic skills for developmental stage classification by receiving feedback from the decision-making process illustrated in Fig. 6. In the future, human and AI collaboration in dentistry will be expected in academic education and clinical practice [13, 46, 47].

This study has some limitations. Although the number of training datasets in this study is much larger compared to that in previous studies in the field of dentistry, it is still small compared to that in other fields. For example, ImageNet consists of 14 million natural images [48], MS-Celeb-1 M has 10 million face images [49], and RadImageNet provides 1.35 million medical images [50]. There may be some room for further improvement of automatic calculation performance by training with a larger dataset. Also, our datasets contain a relatively large number of healthy images. To reduce this bias and to overcome imbalanced datasets, adding the public datasets such as Tufts Dental Database [51] or federation learning across multiple medical institutions [52] could be solutions to be consider.

Another limitation is that our datasets lack metadata like chronological age or sex because of ethical reasons. In particular, since the metadata of sex and race are important factors for dental age estimation, they are necessary to evaluate the difference between our results and further studies in which metadata are available. Additionally, if the metadata of age are available, our model can be modified to calculate not only dental age but also chronological age, which is useful in forensic science [45]. Thus, a large-scale dental image dataset, which has metadata and is annotated by experts, is expected to help in developing successful AI models in dentistry.

## Conclusion

In this study, we achieved automatic dental age calculation with a clinically acceptable error compared to manual calculation by human experts using two-stage deep learning with high accuracy in dental germ detection and developmental stage classification. Dental age is crucial for treatment planning in pediatric and orthodontic dentistry, and our method supports faster dental treatment planning than that with manual calculation.

## Data Availability

The datasets generated or analyzed during the current study are not publicly available to protect patient privacy but are available from the corresponding author on reasonable request.
